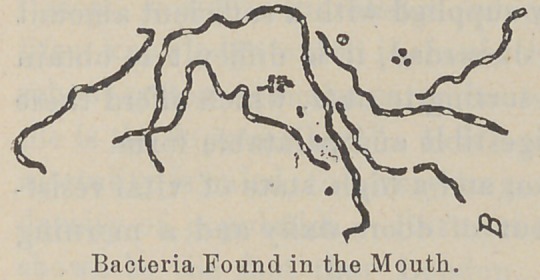# Are We to Be a Toothless Race?

**Published:** 1899-03

**Authors:** J. H. Kellogg


### SELECTIONS.

### Are We to be a Toothless Race?

BY J. H. KELLOGG, M.D.

Abstract of a lecture delivered in the parlor of the Battle Creek Sanitarium.

Twenty-five years ago dentists were not nearly so numerous as they are to-day. They used to travel from town to town, carrying their outfits with them, in a manner similar to that of Methodist itinerant clergymen in the early history of that denomination. They would do such jobs of dentistry as they could find in one town, and then go to another, with difficulty earning a livelihood. But at the present time this profession is a very lucrative one; dentists are growing rich. So many people are putting their gold into their teeth that, as some one has suggested, the gold-mines of the future are likely to be found in the cemeteries of the present day.

This decay of teeth is not a local accident or a matter of mere local interest; it is an indication of constitutional decay—of the decay of the human race. A horse-dealer would not buy a horse that had decayed teeth; he would know that that horse was losing his vitality and growing weak; and yet people offer themselves to the world as being strong and vigorous when they have scarcely a sound tooth in their heads. We find young people from twenty-two to twenty-five years of age getting married—and without a sound tooth. Such people are not fit to be fathers and mothers. Their constitutions have already begun to decay, and their decayed teeth are an evidence of that constitutional deterioration.

Teeth decay just as apples, potatoes and other fruits and vegetables decay, just as dead animals decay—through the action of germs. Germs, when allowed to take up their abode in the mouth and to develop in large numbers form colonies upon the teeth. They accumulate in the mouth, and grow there in the form of patches. In the morning the teeth will feel rough, and will be covered with a yellow or whitish slime. This roughness is due to germs. They come from the food, the air, and the water, and feed upon the remnants of food which they find in the mouth and between the teeth.

The saliva and the mucous membrane have power to destroy germs to some extent. The experiment has been made of planting deadly germs upon the septum of the nose, and in three hours they were killed by the mucous membrane. Healthy living tissue has the power to kill germs. When the mouth is in a clean, wholesome condition, germs cannot obtain a foothold there; but when fragments of meat and other particles of food are left in it, they become hotbeds for germs which feed upon these fragments of food, develop and prey upon the teeth. Uncleanliness of the mouth is, therefore, one reason for the development of germs. Another cause of germs in the mouth is the loss of ability in the body to destroy them, and the cause of this lowered resistance of the body is, as a rule, poor digestion. A weakened body that is losing its resistance to germs manifests this loss of resistance as quickly in the teeth as in any other part of the body, and perhaps more quickly. The premature decay of the teeth, then, is always a sign of a weak and enfeebled constitution. Sound teeth are an indication of a sound body.

The first thing a doctor does when a child is brought to him for treatment is to examine its teeth. If a child of ten years has decayed teeth, it will be almost certain to die prematurely. Primitive people and savage tribes live to be very old, and have sound teeth, some even having a third set when very old. In some of the marshes in the northern part of Michigan the skeletons of mammoths have been found with the teeth in perfect condition, yet these mammoths have been dead perhaps two or three thousand years. I have seen a dozen or more mummies in the British Museum with perfectly sound teeth.

Human Teeth Covered with Tartar.

These facts show that teeth ought not to decay; that they ought to be the best preserved of any structures in the body; that they ought to remain intact longer than any other part; and yet in this country we find them decaying earlier than any other structure because of the action of germs and the loss of resistance on the part of the body.

When the teeth are sound, they are so covered with enamel as to be protected from changes of temperature and from injury from other causes. In a natural state, they are strong enough to bite hard substances without difficulty. Being protected by such a coat of mail, as it might be called, how is it possible for them to decay? The decay of the teeth is accomplished in this way: Some of the germs that form colonies in the mouth and upon the teeth secrete a substance which is capable of dissolving the enamel, and then the germs gradually work their way into the teeth. You have probably noticed old crags and rocks upon mountain tops where mosses and lichens have grown, destroying a portion of the hard substance beneath them. These mosses are capable of forming substances which can dissolve the hardest rock, crumbling it and finally disintegrating it. So it is with the germs that grow in the mouth; they are capable of gradually dissolving the enamel and crumbling it off, after which they work their way down into the teeth.

Human Teeth Injured by Tartar.

The teeth usually begin to decay in the hidden parts, not upon the surface. Decay most frequently begins between the teeth, because portions of food are caught and held there, and there the germs grow unmolested. Between thirty and forty different kinds of germs can live in the mouth, a large number of which are capable of attacking and destroying the teeth.

There is a popular idea that sugar, candies and other sweets cause dental decay. This subject has been much discussed, and authorities are not entirely agreed upon it. While some people say, "I have eaten quantities of candy, and my teeth are sound," others say that candy has destroyed their teeth. It seems to me to be a question of digestion rather than of sweets. There are certain persons who can eat candy with impunity, because they have the ability to resist that particular cause of indigestion; but in other cases it produces indigestion, and indigestion diminishes vital resistance, thus favoring the attack of germs and dental decay.

Another cause of the premature decay of the teeth is amylaceous or farinaceous dyspepsia, or starch indigestion. This is becoming an almost universal disease among the American people.

Bacteria Found in Putrefying Matters.

It greatly lessens the vital resistance of the body in every part, including the teeth. Germs being more numerous in the mouth than in any other part of the body, their effects are produced in the mouth and upon the teeth to a greater degree than elsewhere when the teeth are left covered with fragments of meat and other debris.

The skin soon becomes diseased when dirt is allowed to accumulate upon it. It is well known to skin specialists that dirt is a prime cause of skin disease. If one should allow the skin to become as dirty as the teeth sometimes do, nothing could prevent the action of germs upon the skin, and it would soon succumb to their attacks. So it is not a strange thing that the teeth succumb to germs.

Bacteria which Infest the Mouth and Alimentary Canal.

One of the results of the growth of germs in the mouth is a coated tongue. If a person has a coated tongue, he is sure, sooner or later, to have decayed teeth. Ice-cream is considered by many to be bad for the teeth; others are of the opposite opinion. Even some dentists try to show that ice-cream is not injurious to the teeth because it does not destroy the enamel. But while it may not hurt the teeth directly, it injures the stomach, produces biliousness and a coated tongue, thus lowering the vital resistance of the body and exposing the teeth to destructive germ-action.

Another important cause of the decay of the teeth is the failure to furnish proper food for their nourishment. The popular idea is that there are not enough salts in our food; but the difficulty does not consist in a lack of these elements in our food, but rather in a lack of ability to absorb and digest them. Grains and nuts contain an abundant supply of salts. It is the acid of the stomach which prepares these salts for absorption; and when this is not present in proper quantity, we are unable to digest and absorb them, and thus become subject to softening of the teeth from lack of nutrition.

Bacteria of Dental Caries.

It is supposed that phosphate of lime and carbonate of lime are necessary for the teeth, because, when the teeth are burned, these elements are left. It is thought that we do not get a sufficient quantity of these elements in our food, hence doctors prescribe doses of them for the teeth. But any one who wishes to take a dose of phosphate of lime for the nourishment of the teeth only needs to eat an extra slice of graham bread or a granose biscuit.

Last year I made a study of a hundred cases of persons, some of whom had hypopepsia and others hyperpepsia. Careful examination was made of their stomach fluids and of their teeth, and it was found that those who had hyperpepsia had twice as many teeth left as those who had hypopepsia. The conclusion from this examination was this: in hypopepsia the stomach has lost the power to make gastric juice and to destroy germs, hence this condition is more favorable to the decay of the teeth than hyperpepsia, in which there is an excess of hydrochloric acid in the stomach, therefore a good ability to destroy germs and to disinfect the food.

There is no doubt that the use of flesh food is one of the chief causes of dental decay. The reason is that little particles of meat get between the teeth, and encourage the growth of destructive germs. The germs that destroy teeth are the same as those that cause the decay of flesh. It is better not to have dead things of any sort lying around in the mouth for the subsistence of germs, but to keep it entirely clean and free from fragments of dead animals; and that can be most easily accomplished by putting nothing of the sort into the mouth. The monkey is a vege-

Teeth of a Healthy Cow.

tarian, and has sound teeth, so I recommend persons who wish to have healthy teeth to live on the monkey's diet—fruits, grains and nuts; there is nothing healthier.

A vegetarian diet is, without question, conducive to the development and preservation of the teeth, because, first, it excludes meat; and secondly, it gives us food in proper condition for the use of the teeth—food that requires thorough mastication. The teeth need exercise, and a vegetarian diet gives them healthy and normal exercise. Flesh food does not usually require much mastication. Our teeth are not adapted to eating flesh, but to the cutting, crushing and grinding of grains, fruits and nuts.

People generally eat too much soft food. If we could banish spoons and forks from our tables, so as to be compelled to take all our food hard and dry and from the hand, as we now take bread, it would be much better for the race. I am satisfied that lack of use is one cause of the premature decay of the teeth.

In order to preserve the teeth, it is necessary to use them.

In the famous Mammoth Cave, there are fishes that have no eyes, or mere rudiments of eyes. They have gradually wasted away from non-use. There are strong indications that the people of the United States are in a fair way to lose their teeth in the same manner, from lack of use, and to become in course of time a toothless race. In New York City it has been found that the teeth of the cows of the distillery dairies, which live on distillery slops that do not require mastication, are almost entirely gone. The teeth should be scoured by rubbing against hard food that will polish them. A wagon tire is kept clean and bright by constant movement and by coming in contact with the hard ground; machinery is kept in good condition by constant use and friction. So, when dry food comes in contact with the teeth, it scours them, keeps them clean, and preserves them. I know of nothing better adapted to this purpose than granose, zwieback, or the dry crusts of hard bread.

People are likely to be reckless in regard to losing their teeth. Never lose a tooth if you can avoid it. I have known some people to have sound teeth taken out in order to have some new and handsome "store-teeth" put in. That is the greatest possible mistake. Keep your teeth as long as you can, even if they are not handsome. Get them filled as soon as there is the slightest decay perceptible.

To lose a tooth is to lose a part of one's life. We are not aware how dependent we are upon our teeth for our physical welfare, because we do not stop to think what a beautiful apparatus they form for grinding our food. The mouth is a mill formed upon the "gradual reduction" plan. Our front teeth furnish an apparatus for cutting the food. The side teeth, the cuspids, or cutting teeth, are sometimes improperly called "canine teeth," or "dog teeth," and some people argue that because we have "dog teeth" we must be carnivorous animals. But these teeth are not "canine teeth;" they are made for cutting the food, while the dog's teeth are constructed for the purpose of tearing and "hetcheling" food. Then we have the bicuspids, or small molars, by which the food is made still finer; then the molars, or multi-cuspids, including the "wisdom teeth," which complete the grinding process. So, when we speak of the mastication of a morsel of food, we mean that it is first cut by the front teeth, then passed a little farther back in the mouth to the small molars, or bicuspids, for further grinding; then still farther back to the molars for completion of the grinding process. The food is thus worked farther and farther back in the mouth, and ground up finer and finer until it gets behind the "grinders," or molars, and then it is close to the throat and ready to be swallowed, having been sufficiently masticated.

Teeth of a Cow Fed on Distillery Slops.

While the food is being ground by the teeth, it is the duty of the cheeks to keep it between the teeth, first upon one side and then upon the other, thus giving one side a rest while the other is at work. But in order that this may be done, the upper and lower teeth must fit. If there is a tooth gone, when a coarse particle of food comes along to that place, it drops down into the empty space and is not properly masticated. These coarse portions of food slip into the throat, and are swallowed into the stomach in an improper condition and make trouble. A person suffers from the same cause if his teeth are not in perfect apposition, because, in that case also, the food will slip into the stomach without being properly masticated. Such a person cannot masticate his food sufficiently, unless he chews it all in front, which few are willing to do. If the teeth are defective upon one side, then the person must chew his food altogether on the other side; but this will certainly wear out the teeth on that side, and give rise to new difficulties.

There is another argument for taking proper care of the teeth:

If a tooth is gone upon one side, nature tries to remedy that defect by gradually approximating the remaining teeth; so they keep coming nearer and nearer together until the shape of the jaw is changed. If several teeth are lost, the shape of the jaw is so changed that the face is materially altered. The change produced in the appearance of the faces of elderly persons, in consequence of the loss of teeth, is very marked. Thus defective teeth cause greater or less deformity, altering the appearance and expression of the countenance.

The teeth can be kept clean by the use of pure, soft water—distilled water, if possible—and a brush. These are the only essentials, if one regularly maintains the habit of cleaning his teeth after every meal. Rub vigorously not only the front teeth, but all the teeth, including the back ones, which are the most important; they should be rubbed and polished until they feel smooth to the tip of the tongue. If not perfectly clean, one should have them cleaned by the dentist; and when they are once smooth, take good care to keep them so.

Bacteria Found in the Mouth.

If the teeth have been neglected, it is advantageous to add something, as precipitated chalk, to the brush and water. But do not use the ordinary dentifrices or soap. It is well, ordinarily, to use an antiseptic. The best of all antiseptics is cinnamon oil, or some preparation of this oil. To prepare cinnamon water, take a teaspoonful of cinnamon essence, add four ounces of water, shake thoroughly, and allow to settle. The oil which does not dissolve will settle to the bottom. If the water is then turned off, it will be well saturated with cinnamon. This is a perfect germicide.

The proper course of life for one who wishes to enjoy the use of sound teeth in old age may be summarized as follows:

Live temperately in all things; adopt a dietary of fruits, grains and nuts; eschew meats, cheese, condiments, pickles and all unwholesome foods, so as to preserve sound digestion; eat dry food, and especially thoroughly cooked cereal foods, so as to avoid farinaceous dyspepsia and indigestion; granose, granola, zwieback, and hard-baked unleavened breads of all sorts are to be commended; soups, mushes, gruels, and soft grain preparations are all imperfectly cooked unless they have undergone previous preparation, as in the case of crystal wheat, browned rice, browned wheat, etc.; soft, imperfectly cooked cereal foods are a most prolific cause of indigestion, and hence of general decay, as has already been pointed out. Make free use of nuts and nut products, such as nuttose-C, nuttolene, bromose, malted nuts, etc. The system must be constantly supplied with a sufficient amount of fat, and if animal fats are discarded, it is difficult to obtain the requisite supply without resorting to nuts, which afford these elements in the purest, most digestible and palatable form.

To maintain a good digestion and a high state of vital resistance, take vigorous exercise out of doors daily and a morning cool or cold bath.

## Figures and Tables

**Figure f1:**
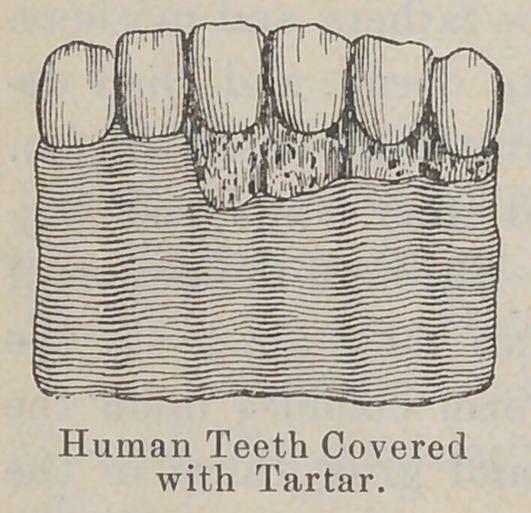


**Figure f2:**
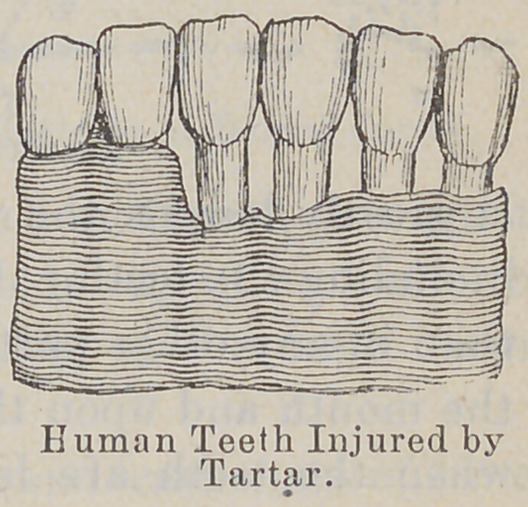


**Figure f3:**
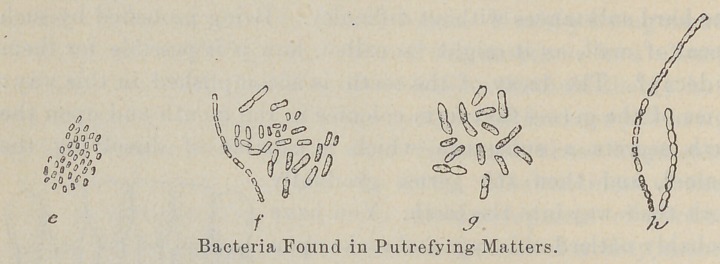


**Figure f4:**
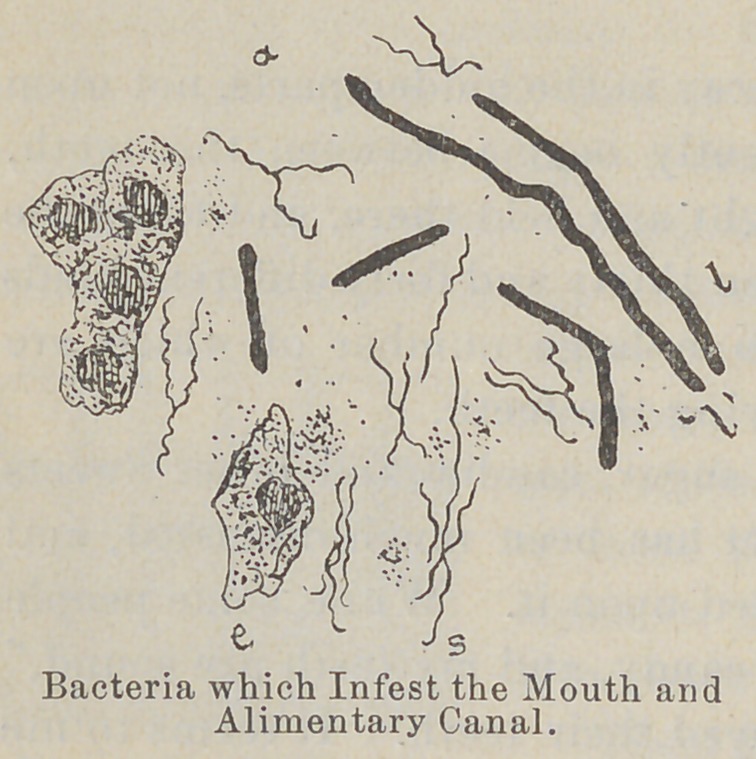


**Figure f5:**
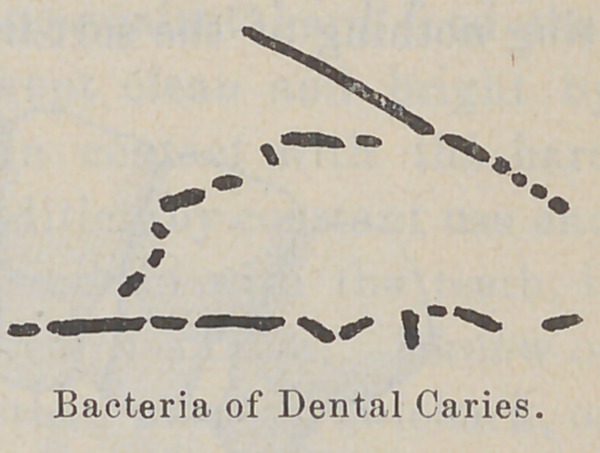


**Figure f6:**
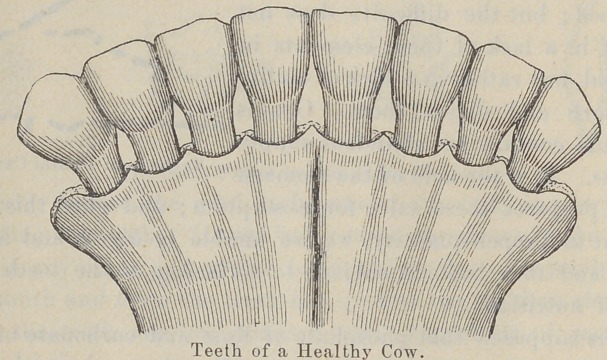


**Figure f7:**
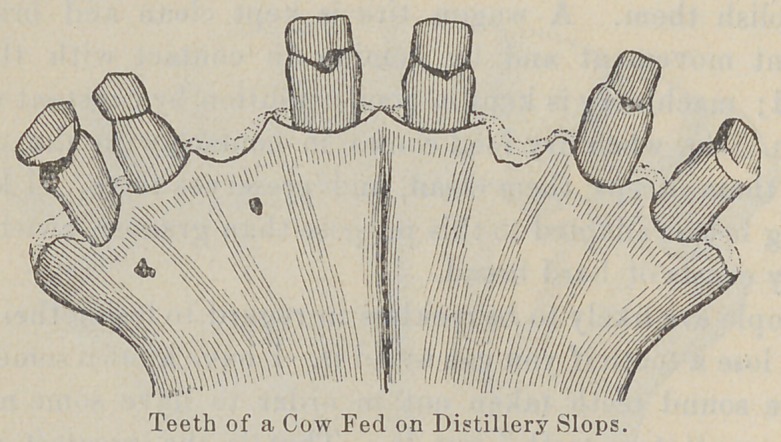


**Figure f8:**